# The Potential Role of NOD2/CARD15 Genotype as a Prognostic Indicator for Bone Marrow Transplantation Outcomes in Patients With Acute Lymphoblastic Leukemia and Acute Myeloid Leukemia: A Systematic Review

**DOI:** 10.7759/cureus.52329

**Published:** 2024-01-15

**Authors:** Leila Ahmadinia, Shahid B Rangrej, Maria Miranda, Christine Shailer, Waleed Ahmed, Victoria Carvalho, Rajni Rathore

**Affiliations:** 1 Basic Sciences, Saint James School of Medicine, Arnos Vale, VCT; 2 Anatomy/Research, Saint James School of Medicine, Arnos Vale, VCT; 3 Pharmacology and Therapeutics, Saint James School of Medicine, Arnos Vale, VCT

**Keywords:** acute myeloblastic leukemia (aml), acute lymphoblastic leukemia (all), prognostic indicator, bone marrow transplantation, nod2-card15 genotype

## Abstract

Hematopoietic stem-cell transplantation (HSCT) has emerged as a groundbreaking therapeutic option for acute myeloid leukemia (AML) and specific subtypes of acute lymphoblastic leukemia (ALL). The prognostic significance of the NOD2/CARD15 gene has been explored alongside various factors, encompassing diverse patient cohorts and gene variants. Siblings and unrelated donors used for stem cell transplantation exhibit significant associations between their genetic variations and graft-versus-host disease incidence. The transplantation of stem cells for leukemia patients involves numerous considerations, including patient survival, relapse rates, disease stage, donor and recipient ages, and compatibility. This study delved into research on the NOD2/CARD15 gene and its mutations to assess its suitability as a screening tool. A comprehensive literature search encompassing PubMed, ScienceDirect, and Google Scholar articles yielded 4,840 articles. After removing duplicates and applying inclusion and exclusion criteria, we narrowed the search results to 876 articles. Subsequent screening of abstracts and titles resulted in the selection of 230 relevant articles. Further exclusion of 198 articles unrelated to the research question led to the scrutinizing of 32 full-text articles, which were assessed against inclusion and exclusion criteria. Emphasis was placed on articles that specifically investigated the role of NOD2/CARD15 as a predictive factor for HSCT outcomes, ultimately resulting in the inclusion of 19 articles in this study. Single nucleotide polymorphisms (SNPs) such as NOD2 and CARD15 have demonstrated their potential as reliable genetic markers for predicting post-transplantation relapse and disease outcomes. Patients positive for these genetic markers have exhibited reduced overall survival and event-free survival and increased transplant-related mortality. Interventions with interferon-gamma and muramyl tripeptide phosphatidylethanolamine have been considered to mitigate the inflammatory effects of these SNPs, thus enhancing the influence of natural killer cells on abnormal cells and potentially extending patient survival. NOD2/CARD15 typing may aid in identifying patients at higher risk for relapse and improving their clinical outcomes after allogeneic stem cell transplant, particularly in ALL patients. However, no remarkable change was observed in AML patients. Additionally, this study underscores the pivotal roles of adaptive and innate immune responses and their interplay in stem cell transplant immunology.

## Introduction and background

Hematopoietic stem-cell transplantation (HSCT) is a breakthrough treatment for acute myeloid leukemia (AML). AML is the most common adult type of leukemia in adults, and acute lymphoblastic leukemia (ALL) is the most common in children [1]. NOD2 and CARD15 determine the outcome of AML and ALL treatments [2]. According to Grube et al., NOD2 is a nucleotide-binding oligomerization domain. CARD15 is the caspase recruitment domain 15 gene [2]. Mayor et al. discussed the development of graft-versus-host disease (GvHD) as one of the main complications of post-HSCT [3].

NOD2 is a protein that is an essential mediator of inflammation and forms a protein complex called inflammasome. NOD2 has a connection with AML and ALL on innate immunity, cell survival, and apoptosis. NOD2 has a critical role in deterring the recurrence of leukemia after a stem cell transplant. Also, the receptors similar to NOD2 recognize microbial antigens. Therefore, NOD2 is essential in the incidence of infections and their outcome of AML transplantation [3].

CARD15 is a gene that encodes for NOD2. CARD15 was studied to determine leukemia relapse and mortality rate of patients. The prognostic role of NOD2/CARD15 was analyzed amongst various factors, including different cohorts and variants of the gene. Research shows that donors for stem cell transplantation were siblings or unrelated and that gene variations had a significant association with GvHD [3].

GvHD influences the mortality rate of patients after stem cell transplantation. GvHD has traditionally been categorized into acute and chronic forms, with the distinction made based on the timing of onset, typically using a threshold of 100 days after transplantation. The development of GvHD after day 100 is considered chronic, and before the 100 days is considered acute [4]. Donor and recipient genotypes contribute to the severity of GvHD. Research shows the role of receptors in GvHD to prevent the disease and to preserve the donor response to stem cell transplantation [5].

This systematic review is beneficial for siblings and non-related donors with AML or ALL to determine the predicting factor of the gene in stem cell transplantation. The study is innovative and shines a light on the possibilities of HSCT. This research is essential to add to the body of literature because of the prognosis of the NOD2/CARD15 gene after stem cell transplantation.

## Review

Methodology

To identify the predicting factors of screening NOD2/CARD15 on bone marrow transplantation outcomes in AML and ALL patients, a literature search was done to identify the most relevant scholarly articles. The design of this study is a systematic review. The search was done in PubMed, ScienceDirect, and Google Scholar. The keywords that were used for the search included "NOD2-CARD 15 leukemia," "NOD2-CARD15 genotype bone marrow transplant," "NOD2-CARD15 acute myeloblastic leukemia," " NOD2-CARD15 acute lymphoblastic leukemia," "bone marrow transplantation outcome in acute myeloblastic leukemia patients," "bone marrow transplantation outcome in acute lymphoblastic leukemia patients," "NOD2-CARD15 predicting factor in bone marrow transplant," "screening NOD2-CARD15 for transplantation outcome," "NOD2-CARD15 genotype," "bone marrow transplantation outcome in leukemia patients," "screening patients NOD2-CARD15 before bone marrow transplant," "NOD2-CARD15 screening as predicting factor for bone marrow transplant complications," "NOD2-CARD15 association to post bone marrow transplant complications," and "NOD2-CARD15 reduction in survival post-transplantation." Inclusion and exclusion criteria were applied, and the calculations of the number of results in search terms were done. The inclusion criteria embraced articles related to NOD2/CARD15 as predicting factors for bone marrow transplantation outcomes in patients, articles that were written in English, articles published from 2002 to 2022, articles that reported NOD2/CARD15 as a predicting factor of bone marrow outcome in AML patients, articles that covered adult patients with NOD2/CARD15 with complications of post bone marrow transplant, articles that have provided quantitative and qualitative primary research, as well as articles that have discussed secondary scholarly studies, case reviews, and case studies. The exclusion criteria utilized were associated with the articles that did not relate to the research question and articles that reported no correlation between NOD2/CARD15 as a predicting factor for bone marrow transplant outcome in patients.

The studies used for the systematic review were diverse in publication year, study design, aims, and sample population size. The dominant sources used were a literature review (n = 7), followed by a systematic review (n = 5), meta-analysis study (n = 4), cohort study (n = 1), case study (n = 1), and dissertation (n = 1).

**Figure 1:**
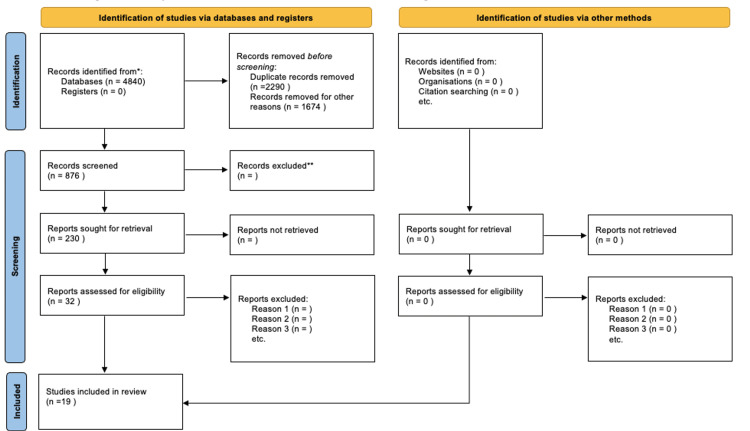
PRISMA flow diagram PRISMA: Preferred Reporting Items for Systematic Reviews and Meta-Analyses.

The flow diagram below (Figure 1) indicates the procedure for the inclusion/exclusion of the studies used in the systematic review. The search on PubMed, ScienceDirect, and Google Scholar found 4840 articles. The removal of duplicates and the initial application of inclusion and exclusion criteria allowed for the summarizing of 876 articles. Screening of abstracts and titles led to the selection of 230 articles. The exclusion of 198 articles unrelated to the research question resulted in the selection of 32 full-text articles that were additionally examined against the inclusion and exclusion criteria. Emphasis was placed on the articles that focused on screening NOD2/CARD15 as a predicting factor for bone marrow transplantation outcomes, which resulted in identifying 19 articles used in this study.

With thorough evaluation and analysis of the presented studies and dissertation, 19 articles were chosen to be included in our systematic review. These articles support our research question and proposal. Combined information in these articles was analyzed to demonstrate how screening the NOD2/CARD15 genotype predicts the bone marrow transplantation outcome in AML and ALL patients. The research data and qualitative, quantitative, and graphic representations in these 19 articles further strengthen the validity of our proposal. The data are focused and dissected further in our results section.

Results

Some of the many considerations when transplanting stem cells in treating AML patients include the length of survival time, relapse likelihood, the stage of the disease, the age of the donors and recipients, and matching. Several studies have been done to determine if the NOD2/CARD15 gene can be used to provide information to improve the outcome of these transplants. We looked at research into the NOD2/CARD15 gene and its mutations to evaluate if this could be useful as a screening tool. Table 1 shows a summary of the findings, and the following Tables 2, 3 show comparable information from two studies reviewed in our paper [3,6].

**Table 1:** A comparison of the results published on NOD2 genotype and hematopoietic stem cell transplant outcome HSCT: hematopoietic stem-cell transplantation; allo-SCT: allogeneic stem cell transplant; GvHD: graft-versus-host disease; GVL: graft-versus-leukemia; allo-HSCT: allogeneic hematopoietic stem cell transplantation; IFN-γ: interferon-gamma; AML: acute myeloid leukemia; SCT: stem cell transplant; BO: bronchiolitis obliterans; cGvHD: chronic graft-versus-host disease; SNP: single nucleotide polymorphism; BMT: blood or marrow transplant; AL: acute leukemia; aGvHD: acute graft-versus-host disease.

Author (publication year)	Study design	Summary of results	Total sample size
Grube et al. (2015) [2]	Cohort study	The aim of this study is to demonstrate that NOD2 SNP13 has an impact on the pathophysiology of severe infectious complications and is an independent risk factor for the development of septic shock after allo-SCT.	165
Mayor et al. (2007) [3]	Cohort study	Identify how NOD2/CARD15 is implicated in immune function and disease relapse post-HSCT.	392
Heidegger et al. (2014) [5]	Systematic review	The purpose of this article is to summarize current knowledge about the role of innate immunity in the pathogenesis of GvHD and GVL following allo-HSCT.	N/A
Holler et al. (2008) [6]	Cohort study	T cell depletion interacts with the effect of NOD2/CARD15 on allogeneic stem cell transplant outcomes.	342
Buteyn et al. (2020) [7]	Meta-analysis study	These results suggest that NOD2 activation, primed by IFN-γ, may provide a novel therapeutic option for AML, providing a survival advantage in AML animal models.	N/A
Granell et al. (2006) [8]	Systematic review	Demonstrate that NOD2/CARD15 variants have a deleterious effect on clinical outcomes in T-cell-depleted allogeneic SCT.	156
Holler et al. (2004) [9]	Literature review	The study aims to indicate a significant role of monocyte/macrophage in the pathophysiology of GvHD and strongly suggests a future risk assessment or even donor selection through NOD2/CARD15 typing.	169
Mayor et al. (2010) [10]	Literature review	To prove that NOD2/CARD15 has been correlated with detrimental outcomes post stem cell transplantation.	22
Hildebrandt et al. (2008) [11]	Systematic review	This study aims to demonstrate that multivariate analysis proved recipient but not donor NOD2/CARD15 variants to be a novel independent risk factor for BO development, and NOD2/CARD15 typing may help identify patients at increased risk for bronchiolitis obliterans complications.	N/A
Crossland et al. (2020) [12]	Literature review	The aim of this study is to demonstrate that the prognosis or prediction of outcome for cGvHD can use many novel sources of potential markers that have shown promise.	N/A
Holler et al. (2006) [13]	Systematic review	This study aims to perform an analysis focusing only on HLA-identical sibling transplantations, including patients from the first cohort and a larger, second, and independent cohort of patients to address the impact of NOD2/CARD15 SNPs on long-term transplantation.	N/A
Holler et al. (2010) [14]	Literature review	The aim of this study is to demonstrate that GvHD is the most serious complication of allogeneic stem cell transplantation (SCT) and results from an activation of donor lymphocytes by recipient antigen-presenting cells (APCs).	N/A
Mayor et al. (2012) [15]	Literature review	This study aims to review the existing literature, summarize current theories as to why the data differ on the impact of NOD2 on HSCT, and suggest possible mechanisms by which the SNPs affect HSCT outcomes.	N/A
Karaesmen et al. (2017) [16]	Meta-analysis study	The aim of this study is to emphasize the peril of pursuing candidate approaches and the importance of adequately powered tests of unbiased genome-wide associations with BMT clinical outcomes, given the ultimate goal of improving patient outcomes.	2888
Madrigal et al. (2007) [17]	Literature review	This study suggests that prospectively typing AL patients for HLA-DPB1 and NOD2/CARD15 SNPs will allow the prediction of disease relapse, aGvHD, and cGvHD.	N/A
Sioud et al. (2009) [18]	Experimental study	Demonstrate that the NOD2 signaling pathway alone was found to be sufficient to drive BM CD34 cells to secrete cytokines and differentiate into innate effector cells.	N/A
AL-Battawi S et al. (2018) [19]	Literature review	This study aimed to evaluate the impact of cytotoxic T-lymphocyte antigen-4 (CTLA-4) and caspase recruitment domain 15 (NOD2/CARD15) gene polymorphisms on the incidence and severity of aGVHD and cGVHD following allo-HSCT.	N/A

**Table 2:** Comparison of GvHD, relapse, and overall survival in patients with WT or SNPs Reference [3]. OS: overall survival; DFS: disease-free survival; TRM: treatment-related mortality; GvHD: graft-versus-host disease; HvGD: host-versus-graft disease; N/C: no change; SNP: single nucleotide polymorphism; WT: wild type.

NOD2-CARD15 in donor	NOD2-CARD15 in patient	Overall survival	DFS	Relapse	GvHD
WT	WT	44%	40%	32%	-
WT	SNP	22%	19%	54%	GvHD
SNP	WT	N/C	-	N/C	HvGD

**Table 3:** Comparison of GvHD, relapse, and overall survival in SNP patients with AML or ALL Reference [6]. OS: overall survival; DFS: disease-free survival; TRM: treatment-related mortality; GvHD: graft-versus-host disease; HvGD: host-versus-graft disease; N/C: no change; SNP: single nucleotide polymorphism; WT: wild type; AML: acute myeloid leukemia; ALL: acute lymphoblastic leukemia.

Disease	NOD2-CARD15 in donor	NOD2-CARD15 in patient	Overall survival	Relapse	GvHD
ALL	SNP	SNP	Decrease	N/C	Increase
AML	SNP	SNP	N/C	N/C	N/C

Of particular note, the treatment-related mortality (TRM) rate in AML was unaffected by single nucleotide polymorphisms (SNPs) in sibling donors or recipients [3]. The overall survival (OS) and disease-free survival (DFS) decreased with the presence of SNPs in wild-type (WT) pairs, and the rate of relapse increased substantially [3].

Holler et al. (2008) found some differences to the above when looking at unrelated donors (UD), with the presence of SNP13 in donors being the most significant difference in TRM, almost double than that of non-SNP survival [6]. They found no other notable differences in the effects of the other results (OS and DFS) when comparing UD and sibling donors except for a substantial variation in GvHD [6,7]. As their study was heavily composed of AML patients (74% AML vs. 50% other) [3], the results may have been less reactive than in ALL and other blood disease patients [6]. Further investigations concluded that T-cell depletion was the main difference between the two studies.

NOD2/CARD15 is known to signal natural killer (NK) cells, which may trigger a response to attack acute leukemia [6,7]. First in an in vitro then an animal in vivo study, treatment with a combination of IFN-γ (interferon-gamma) and MTP-PE (muramyl tripeptide phosphatidylethanolamine - a synthetic molecule that binds to NOD2) on AML brought on a cytokine response that activated NK cell production [7]. Using both treatments together decreased the disease and increased OS in mice vs. single-treated and untreated mice [7].

T-cells are lymphocytes that are active in the immune system. They may be depleted in donors before stem cell transplant to reduce the potential for GvHD in the recipient by reducing donor cells that attack the recipient's healthy and infected cells.

Results of one study on the effect of NOD2/CARD15 variants in recipients of transplant after T-cell depletion are shown in Table 4 [8].

**Table 4:** Mortality and survival in patients with WT and SNPs Reference [8]. SNP: single nucleotide polymorphism; WT: wild type; DFS: disease-free survival.

Variants present	DFS	Non-relapse mortality
SNP	17%	49%
WT	48%	23%

A study split SNPs according to their presence in either recipients or donors or both. There was an increased risk of GvHD in patients with donor SNPs, a remarkable rise for both overall and gastrointestinal GvHD in pairs with mutations of both patients and donors. As a consequence, the cumulative incidence of one-year TRM increased from 20% in WT patient/donor pairs to 49% in pairs with recipient (P = 0.03) to 59% in teams with donor (P = 0.004), and 83% if donor and recipient revealed both mutations (P = 0.001) [9].

Some studies looked at combinations of SNPs in UDs to see how different mutations and SNPs in the NOD2/CARD15 gene are related to different diseases and the outcome of the HSCT in patients. Haploid type H1 is WT, SNP 5 + 6 is haplotype H2, and SNP 5 + 6 + 8 is H3. This study sorted patients with ALL (45%) and AML (55%) in the same group. However, further studies show no change in the transplant outcome with the presence of SNPs in the NOD2/CARD15 gene in AML patients, and it affects only ALL patients, but the summary of these studies is shown in Table 4. This study is useful in differentiating diseases relating to each polymorphism subtype of NOD2/CARD15 SNPs and has no benefit in clinical use.

In this study, the OS in the presence of SNPs in either patients and UDs in both AML and ALL patients after eight years was 28%, and relapse was 41% [10].

When compared to the outcomes with single SNPs 8, 12, and 13, there was a distinct advantage of combining haplotypes. Also note that 60% of WT were found to have at least one SNP [10], as illustrated in Table 5.

**Table 5:** Frequency, relapse, and survival (overall and disease-free survival) in haplotypes of NOD2/CARD15 Reference [10]. SNP: single nucleotide polymorphism; WT: wild type; OS: overall survival; DFS: disease-free survival.

	WT (H1/H1)	SNP (H2/H2) homozygous	SNP (H3/H3) homozygous	WT/SNP (H1/H2) heterozygous	WT/SNP (H1/H3) heterozygous
Frequency	70.8%	21.1%	4.6%		
OS (1 year)	55%	67%	-	41%	29%
DFS (4 years)	46%	60%		33%	29%
Relapse (1 year)	25%	60%		32%	50%

The NOD2 gene has an allele mutation (3020insC) that signifies an increased risk of cancer, i.e., 25-35% higher than people without it [5]. NOD2/CARD15 connects to chromosome 16q12, which also can be a marker for a poorer prognosis in laryngeal carcinoma. A person with leukemia may have a coexisting second cancer, particularly in the larynx, head, and neck [5].

Complications post allogeneic transplantation include acute graft-versus-host disease (aGvHD), chronic graft-versus-host disease (cGvHD) [2], pulmonary complications such as bronchiolitis obliterans (BOS) [11,12] and pneumonia [13], and infections such as septic shock [2]. When NOD2 SNPs were studied regarding the development of septic shock, only the donor SNP13 was found to be a significant and independent risk factor for the increased incidence of septic shock [3]. Pulmonary infections and pneumonia deaths were higher in recipients with variant SNP8 and SNP13 than those with WT NOD2/CARD15 [13]. A higher susceptibility toward developing BOS and death was more significant in donor-recipient pairs with SNP variants than in those without [11,12].

The highest risk of severe complications and death after stem cell transplantation is GvHD [2,14]. Acute (aGvHD) disease development is within 100 days post-transplant, and chronic (cGvHD) after that [2]. There has been much research into the NOD2/CARD15 gene and SNPs and their relationship with GvHD. Many studies found that SNP variations vs. WT pairs showed an increase in relapse and a decrease in OS but not in AML patients [6,8,13,15]. In 2018, Amini et al. found that SNP13 was the only NOD2/CARD15 variant to show increased aGvHD occurrence but not severity [2].

With new comparative abilities (DISCOVeRY-BMT from GWAS: Determining the influence of susceptibility conveying variants related to one-year mortality after BMT, genome-wide association study) and many studies to draw from, a publication in 2017 presented an updated opinion of several genes and SNPs and their effects on bone marrow transplant survival [2]. The patients had ALL, AML, and myelodysplastic syndrome. Donors were unrelated, and two cohorts were used. Results showed no association of NOD2/CARD15 SNPs with OS or TRM when analyzed with the DISCOVeRY-BMT method. Indeed, the only variant that showed any potentially damaging or disease-stimulating effect was SNP12 [2]. These results point to the prior association results as false positive, encouraging studies into alternative therapies and individualized treatment.

Discussion

HSCTs are correlated to worse outcomes when associated with specific SNPs [11,16,17] after transplantation [3]. The current study hypothesizes that genes NOD2/CARD15 are genetic markers for post-transplantation and that these SNPs may give better correlation and provide linkage disequilibrium (LD) with other populations. These results helped determine outcomes in HSCT in UD-HSCT between 1996 and 2005 in centers in the United Kingdom with patients diagnosed with either AML or ALL [3]. During this time, myeloablative conditioning, T-cell depletion, and most protocols using in vivo alemtuzumab improved event-free survival (EFS) and OS. Individuals who underwent bone marrow transplants demonstrated that SNPs 5 and 6 had the highest OS after one year compared to those with H3 and SNPs 5, 6, and 8 positive. Haplotypes also influenced EFS, with those that were positive for SNPs 5 and 6 having the best chance of an EFS. Individual donor haplotype combinations significantly influenced the probability of disease relapse. Unique haplotypes may have been significantly correlated to the risk of disease relapse; however, haplotypes had no significant effect on non-relapse mortality, such as aGvHD. Multivariate analysis determined significance through the relative risk in different contexts such as OS [6]. More research is required to explore the interrelations of additional polymorphic positions to enhance outcome predictability in treatments like HSCT-UD.

HSCT using UDs has become increasingly popular in treating patients with acute leukemia. The recent discovery of human leukocyte antigen (HLA)-DPB1 may change the face of HSCT with its influence on post-transplantation. Madrigal et al. have demonstrated an increased risk of relapse with HLA-DPB1 and NOD2/CARD15 genotypes matching for transplantation. When researchers decided to control for both genes, not surprisingly, there was an increase in disease relapse. Even though there was increased GvHD, there was still a lower incidence of relapse and better OS. As a result, the preferred pairing would be a mismatched HLA-DPB1. Genotyping of the aforementioned may help predict disease relapse and select a more suitable donor for treatment in AML or ALL with stem cell transplants [17].

Another type of treatment in allogeneic stem cell transplants is increasingly being used as a way to treat individuals suffering from forms of leukemia. SNPs in the NOD2/CARD15 gene have been shown to harm patient outcomes in states of abnormal immune response. GvHD has also demonstrated increased incidence in those with the SNPs above. These findings support that NOD2/CARD15 is more influenced by the host's innate immune defenses than the adaptive immune response typically produced by immune cells in the thymus and bone marrow [17].

GvHD is the main limiting complication of allogeneic HSCTs and proves to create problems since it is the leading treatment for leukemia. Innate immune responses are often associated with the innate pattern-recognition receptors (PRRs), such as toll-like and nod-like receptors in bacteria, which are involved in acute GvHD. Chemotherapy has been proven to disrupt immune responses, resulting in microbes activating host immune cell receptors to produce inflammatory molecules and abnormal immune cell reactions. This displays that traditional therapy has limitations in beneficial patient outcomes, which may serve better as an adjunct. The ultimate goal in HSCT treatment is to preserve T-cell response to malignancy while protecting the patient from relapse. Studies showed that T-cell depletion of CD34+ in allograft cells had reduced occurrences of GvHD [7]. Future research should focus on PRR signaling to spare innate host defenses and maximize eradicating malignancy, which would be the best next step in treating leukemia.

The graft-versus-leukemia (GVL) effect may contribute negatively to patient outcomes with the WT gene product, so genotyping before transplantation can help guide clinical management. Survival of patients has shown to be significantly reduced when receiving transplantation from UDs and those that possess the NOD2/CARD15 SNPs. Patients with WT SNPs may be unable to mount a sufficient immune response to combat the GvHD or GVL by blunted cellular host defenses. SNPS within NOD2/CARD15 of recipients showed decreased free survival and a higher incidence of relapse but no increase in TRM of transplants from UD [6]. Future treatment can consider T-cell depletion as a possible mode of treatment in those with AML to adapt prophylactic strategies based on patients' genotypes, track manifestation of disease relapse, and possibly develop cancer as a comorbidity.

Innate immune cells express surface pattern-recognition receptors, which have been shown to upregulate factors activated in cancer treatment [7]. Similarly, NOD2 is a surface pattern-recognition receptor that controls inflammatory signals in response to invading bacteria and plays a role in acute myeloid leukemia by inducing the death of abnormal cells. Treatment with IFN-γ in unregulated NOD2 involves enzymes that perpetuate downstream cellular signaling for leukemic blast cells to die upon treatment with MTP-PE, a ligand for NOD2 [7]. This increased activation of NK cells, which contributed to relieving disease burden and prolonged survival in patients. By understanding the mechanisms of innate immunity, there can be improved disease management in hospital settings.

NOD2 alterations may also be a predisposing factor for cancer development, such as laryngeal carcinoma, that may worsen patient outcomes by compounding the disease burden of leukemia [18]. Genetic testing for bone marrow transplantation may be added to clinical management protocol to prevent relapse or occurrence of new cancer. Treatment targeted at isolating NOD2/CARD15 can improve OS to reduce the disease burden upon the patient.

Treatment targeted toward bone marrow (CD34+) cells can stimulate toll-like receptors (TLR) to produce cytokines to fight infection or cancer. Bone marrow cells such as CD34+ cells express NOD2, and stimulation by muramyl dipeptide (MDP) leads to inflammatory cell differentiation that is helpful in the replenishment of healthy white blood cells. NOD2 stimulation on CD34+ cells also enhances host immunogenicity by producing defense proteins to help fight AML. This can be groundbreaking for leukemia treatments since immature cells can also be stimulated to enhance innate immune cells for defense against pathogens and graft rejection. Disease occurrence can be modified in patients receiving bone marrow transplants by activating immunity in bone marrow cells to adapt and develop a brand-new immune system [19].

There needed to be more research on UD-HSCT, which reduced assumptions that can be made on whether or not there was a substantial difference between related donors in EFS, OS, and/or TRM. Also, there needed to be more data analysis on haplotype combinations related to HLA types' influence on disease post-transplantation. Moreover, the literature shows an established understanding of immunological mechanisms of treatments for leukemia; however, there was little mention of controlling for age as a possible confounding in those receiving bone marrow transplants.

HSCT research has a strong future with the implementation of comparative analysis for genotypes of other potential loci that may be involved in disease relapse. Exploring the potential risks of other comorbidities may help drive treatment for AML to enable physicians to control better diseases such as GvHD that may create compounding complications for the patient.

## Conclusions

Our review of 19 extensive cohorts and other studies suggests that NOD2/CARD15 mutations may be a novel risk factor for developing relapse in allogeneic stem cell transplant recipients for acute leukemia. These findings indicate potential utility in future risk assessments before transplantation, although there is some variability in the results among different studies. Additionally, the impact of NOD2 variants on HSCT outcomes has yet to be a consensus among the studies, with potential common mechanisms of SNP-induced functional irregularities differing in their effects between groups.

The significance of donor source in these disparities is less pronounced, as advances in transplantation techniques have equalized survival prospects between well-matched unrelated and related donor HSCTs. Personalized medicine, incorporating recipient and donor characteristics, HLA matching, and non-HLA genetics, could be vital in improving outcomes post-transplantation. Ultimately, the data emphasize the potential of NOD2/CARD15 typing to identify patients at greater risk for relapse and enhance their clinical outcomes after allogeneic stem cell transplantation, reinforcing the pivotal roles of adaptive and innate immune responses and their interplay in stem cell transplant immunology.
